# Determination of the strong coupling constant $\alpha _s \left( m_Z \right)$ from measurements of the total cross section for top–antitop-quark production

**DOI:** 10.1140/epjc/s10052-017-5340-5

**Published:** 2017-11-18

**Authors:** Thomas Klijnsma, Siegfried Bethke, Günther Dissertori, Gavin P. Salam

**Affiliations:** 1 Institute for Particle Physics, ETH Zurich, Zurich, Switzerland; 2 Max-Planck-Institute of Physics, Munich, Germany; 3 CERN, Theoretical Physics Department, 1211 Geneva 23, Switzerland; 4 CNRS, UMR 7589, LPTHE, 75005 Paris, France

## Abstract

We present a determination of the strong coupling constant $\alpha _s \left( m_Z \right)$ using inclusive top-quark pair production cross section measurements performed at the LHC and at the Tevatron. Following a procedure first applied by the CMS Collaboration, we extract individual values of $\alpha _s \left( m_Z \right)$ from measurements by different experiments at several centre-of-mass energies, using QCD predictions complete in NNLO perturbation theory, supplemented with NNLL approximations to all orders, and suitable sets of parton distribution functions. The determinations are then combined using a likelihood-based approach, where special emphasis is put on a consistent treatment of theoretical uncertainties and of correlations between various sources of systematic uncertainties. Our final combined result is $\alpha _s \left( m_Z \right) =0.1177^{+0.0034{}}_{-0.0036{}}$.

## Introduction

The strong coupling constant of Quantum Chromodynamics (QCD), $\alpha _s$, is, together with the quark masses, the main free parameter of the QCD Lagrangian. It enters into every process that involves the strong interaction and is the fundamental parameter of the perturbative expansion used in calculating cross sections for processes with large momentum transfers.

The strong coupling is a function of a renormalisation scale $\mu$. Its dependence on $\mu$ is governed by renormalisation group equations [1, 2], however, its value at a given reference scale must be determined from experimental data. The current world average value for the coupling evaluated at the *Z*-boson mass scale, $\alpha _s \left( m_Z \right)$, as determined by the Particle Data Group (PDG), is $0.1181\, \pm \, 0.0011$ [3]. The world average incorporates information from a wide variety of experimental data and of methods to deduce $\alpha _s$ from that data. It requires at least next-to-next-to-leading order (NNLO) accuracy in the perturbative expansions that are used.

Even with the $1\%$ precision that is quoted by the PDG, the uncertainty on $\alpha _s$ contributes significantly to uncertainties on physical predictions for colliders. For example, it leads to about $2\%$ uncertainty on the gluon-fusion Higgs cross section, comparable with the largest of any of the other individual uncertainties [4]. Furthermore, while the bulk of the evidence points to values of the strong coupling that are compatible with $\alpha _s \left( m_Z \right) \simeq 0.118$, including precise lattice-QCD-based determinations, e.g. [3, 5–7], there are a handful determinations with small quoted uncertainties that suggest $\alpha _s \left( m_Z \right)$ values that are several standard deviations below the world average. Notable cases are those from the thrust and C-parameter distributions in $e^+e^-$ collisions, which yield $0.1135 \pm 0.0011$ and $0.1123 \pm 0.0015$, respectively [8, 9],^1^ or the ABMP PDF fit [11], $0.1147\pm 0.0009$.

Of the various NNLO determinations of the strong coupling, so far only one is based on hadron-collider data, using a measurement of the top-quark pair production cross section ($\sigma _{t\bar{t}}$) performed by the CMS Collaboration at a centre-of-mass energy $\sqrt{s}=7\,$TeV [12]. It yields $\alpha _s \left( m_Z \right) = 0.1151^{+0.0028}_{-0.0027}$. This extraction is intriguingly placed between the world average and the outlying low $\alpha _s$ extractions, albeit compatible with both. However, it is based on a single, early and now outdated measurement of $\sigma _{t\bar{t}}$. It is of interest, therefore, to examine how it is affected by more recent precise measurements by the ATLAS and CMS Collaborations at CERN’s Large Hadron Collider (LHC) [13–17] as well as by a combination of measurements from the D0 and CDF Collaborations at the Tevatron [18].

In the course of our discussion, we will encounter issues related to the treatment of theoretical uncertainties and the choice of the parton distribution function (PDF) set that are of relevance more generally in the determination of the strong coupling and other fundamental constants (e.g. the top-quark mass) from collider data. Such studies may become increasingly widespread in the coming years, given the recent rapid progress in NNLO calculations, e.g. for vector-boson (e.g. Refs. [19, 20]) and inclusive jet $p_t$ distributions [21] at hadron colliders and jet $p_t$ distributions in Deep Inelastic Scattering (DIS) [22].

## Determination of $\alpha _s$ from $t\bar{t}$ cross section measurements

### Theory prediction for the top-pair-production cross section $\sigma _{t\bar{t}}$

Theory predictions for the dependence of $\sigma _{t\bar{t}}$ on $\alpha _s$ are calculated using the program top++2.0 [23]. It provides the computation of the total cross section up to NNLO [24], with possible inclusion of soft-gluon resummation at next-to-next-to-leading logarithmic order (NNLL), as described in Refs. [25, 26].

The predicted cross section is evaluated setting both the renormalisation scale $\mu _R$ and the factorisation scale $\mu _F$ equal to the top-quark pole mass. The theoretical uncertainty associated with missing higher-order contributions is evaluated by independently varying $\mu _R$ and $\mu _F$ up and down by a factor of 2, under the constraint that $\frac{1}{2} \le \mu _R / \mu _F \le 2$. The scale uncertainties are modelled as corresponding to a $68\%$ confidence interval with a Gaussian-shaped uncertainty profile. This choice is more conservative than the (flat) $100\%$ confidence interval that is sometimes taken for scale variations and used, notably, in Ref. [12]. The latter choice leads to a scale uncertainty contribution that is smaller by a factor $\sqrt{3}$ (the ratio of the standard deviations of the two uncertainty profiles). Note that a $100\%$ confidence interval for scale uncertainties is known to be inconsistent with the observation that a significant fraction of NNLO calculations is outside the scale uncertainty interval of the corresponding NLO calculation.^2^

**Table 1:** Cross sections and experimental uncertainties for the $\sigma _{t\bar{t}}$ inputs that we use [13, 14, 16–18]. The LHC beam-energy uncertainties quoted in these references have been scaled down by a factor 6.6 in the light of the recent beam-energy calibration [30], which has a 0.1% uncertainty and coincides with the nominal energy within uncertainties. The original beam-energy-induced uncertainties corresponded to $0.66\%$ [31]. The Tevatron beam-energy uncertainty is sufficiently small (cf. Ref. [32]) that no beam-energy uncertainty is quoted by CDF and D0 in the $t\bar{t}$ cross section measurements. The cross section and uncertainties listed here are adjusted to the top mass corresponding to the latest world average value computed by the Particle Data Group [3], $m_t= 173.2 \pm 0.51 \pm 0.71 \,\mathrm {GeV}$. The “Exp. $m_t \text { unc.}$” column corresponds to the $\delta m_t$ uncertainty discussed in Sect. 2.4, signed such that the upper (lower) uncertainty corresponds to an increase (decrease) in $m_t$

	$\sigma _{t\bar{t}}$ (pb)	Statistical unc. (%)	Systematic unc. (%)	Luminosity unc. (%)	E$_{\text {beam}}$ unc. (%)	Exp. $m_t$ unc. (%)
ATLAS (7 TeV) [16]	182.5	1.7	2.3	2.0	0.3	${}^{-0.2}_{+0.2}$
ATLAS (8 TeV) [16]	242.4	0.7	2.3	2.1	0.3	${}^{-0.2}_{+0.2}$
ATLAS (13 TeV) [17]	816.3	1.0	3.3	2.3	0.2	${}^{-0.3}_{+0.3}$
CMS (7 TeV) [13]	173.4	1.2	2.5	2.2	0.3	${}^{-0.2}_{+0.2}$
CMS (8 TeV) [13]	244.1	0.6	2.4	2.6	0.3	${}^{-0.4}_{+0.4}$
CMS (13 TeV) [14]	809.8	1.1	4.7	2.3	0.2	${}^{-0.8}_{+0.8}$
Tevatron (1.96 TeV) [18]	7.52	2.7	3.9	2.8	0.0	${}^{-1.1}_{+1.4}$

A further choice that needs to be made is whether to include the NNLL threshold resummation for the cross section. This is a procedure that resums terms whose leading-logarithmic (LL) structure is $(\alpha _s\ln ^2 N)^n$, where $N \sim d\ln \sigma _{t\bar{t}}/d\ln s$ and *s* is the squared centre-of-mass energy. When $m_{t\bar{t}}^2/s$ approaches one, i.e. when one approaches the threshold for $t\bar{t}$ production, *N* is proportional to $1/(1-m_{t\bar{t}}^2/s$) and the threshold resummation is a necessity. However, at the LHC and even at the Tevatron, top-pair production is far from threshold and *N* is not especially large: for the dominant gluon–gluon production channel at LHC, $N \simeq 1.4$ for $m_{t\bar{t}} = 2 m_t$ and $\sqrt{s} = 7\,\mathrm {TeV}$; while for the dominant $q\bar{q}$ production channel at the Tevatron, $N \simeq 1.8$. Accordingly, there is debate within the community as to whether threshold resummation is called for. On one hand, one may argue that it brings about terms that have a certain physical meaning. On the other, one may argue that there is no reason why the terms brought by threshold resummation should dominate over other, neglected terms, and therefore it is more consistent to include just the fixed-order contributions, which are known exactly. We will take an agnostic approach to this question, carry out fits with and without NNLL resummation, and then average both the central values and the uncertainties in the two cases in order to obtain our final result.

The theory prediction for $\sigma _{t\bar{t}}$ also depends on a choice of PDF set. Since that choice needs to be related to the data that we fit, we postpone our discussion of the PDF choice to Sect. 2.3.

### Measurements of the top-pair production cross section

Our $\alpha _s$ determination is performed using seven $\sigma _{t\bar{t}}$ inputs, listed in Table 1. The six measurements at the LHC include three updated measurements by the CMS Collaboration at centre-of-mass energies of 7, 8 TeV [13] and 13 TeV [14]. These measurements were performed in the $e \mu$ decay channel,^3^ where the *W*-bosons from the top-quark decays each themselves decay into a charged lepton and a neutrino, one of the *W* decays producing an electron, the other producing a muon. The measurements are based on data collected in the years of 2011, 2012 and 2015, respectively, with integrated luminosities of 5.0, 19.7, and 2.2 fb$^{-1}$. From the ATLAS Collaboration, three similar measurements performed in the $e\mu$ decay channel are included, based on datasets with integrated luminosities of 4.6, 20.3 and 3.2 fb$^{-1}$ for the 7, 8 TeV [16] and 13 TeV [17] centre-of-mass energies, respectively. A seventh input from the Tevatron collider [18] at a centre-of-mass energy of 1.96 TeV is included, which comprises a combination of measurements performed in multiple decay channels from both the CDF Collaboration and the D0 Collaboration.

### Choice of PDF

Several considerations arise in our choice of PDF. Firstly, we restrict our attention to recent global fits that are available through the LHAPDF interface [33]. Secondly, we require that the PDFs should be available for at least three $\alpha _s$ values, so that we can correctly determine the $\alpha _s$ dependence of the cross section in the context of that PDF. These two conditions limit us to the CT14 [34], MMHT2014 [35] and the NNPDF3.0 [36] series. Thirdly, we impose a requirement that the PDF should not have included $\sigma _{t\bar{t}}$ data in its fitting procedure. As should be obvious qualitatively, and as we will discuss quantitatively elsewhere [37], using a PDF with top-data included would bias our fits.

**Table 2:** Top-pair cross section data included in a selection of recent PDF fits. A “$\checkmark$” (“$-$”) indicates that the corresponding $t\bar{t}$ cross section measurement is (is not) included in the PDF fit. The specific sets of 7 and $8\,\mathrm {TeV}$ ATLAS and CMS data used in the fits do not always coincide with those that we list in Table 1. All the PDFs shown here predate the $13\,\mathrm {TeV}$ measurements

	Tevatron	ATLAS (7 TeV)	ATLAS (8 TeV)	CMS (7 TeV)	CMS (8 TeV)
CT14 [34]	$-$	$-$	$-$	$-$	$-$
MMHT2014 [35]	$\checkmark$	$\checkmark$	$-$	$\checkmark$	$\checkmark$
NNPDF30 [36]	$-$	$\checkmark$	$\checkmark$	$\checkmark$	$\checkmark$
NNPDF30_noLHC [36]	$-$	$-$	$-$	$-$	$-$

Table 2 summarises what data has been included in each of these PDF sets, including both the default NNPDF30 set and NNPDF30_nolhc, obtained without LHC data. One sees that the two options that are available to us are CT14 and NNPDF30_nolhc.^4^

**Table 3:** Predicted cross sections and uncertainties for the PDF sets that we use [34, 36], as determined with the Top++ program [23] at a reference value of $\alpha _s^\text {ref}= 0.118$. The results are for $m_t = 173.2\,\mathrm {GeV}$ and the “$m_t \text { unc.}$” column corresponds to the $\delta m_t$ uncertainty discussed in Sect. 2.4, signed such that the upper (lower) uncertainty corresponds to an increase (decrease) in $m_t$

	$\sigma _{t\bar{t}}^{\text {pred}}(\alpha _s^{\text {ref}})$ [pb]	PDF unc. [%]	Scale unc. [%]	$m_t$ unc. [%]	$\displaystyle \frac{ \text {d}\ln {\sigma _{t\bar{t}}(\alpha _s^{\text {ref}})} }{ \text {d}\ln \alpha _s}$
**CT14 (NNLO)**					
LHC (7 TeV)	172.7	${}_{-3.8}^{+4.5}$	${}_{-6.5}^{+4.1}$	${}^{-2.6}_{+2.7}$	2.486
LHC (8 TeV)	246.7	${}_{-3.5}^{+4.0}$	${}_{-6.3}^{+3.9}$	${}^{-2.5}_{+2.6}$	2.404
LHC (13 TeV)	807.3	${}_{-2.7}^{+2.6}$	${}_{-5.6}^{+3.5}$	${}^{-2.3}_{+2.4}$	2.133
Tevatron (1.96 TeV)	7.3	${}_{-2.2}^{+3.4}$	${}_{-5.5}^{+3.8}$	${}^{-2.7}_{+2.8}$	1.757
**NNPDF30_nolhc (NNLO)**					
LHC (7 TeV)	174.8	${}_{-5.0}^{+5.0}$	${}_{-6.5}^{+4.1}$	${}^{-2.6}_{+2.7}$	2.247
LHC (8 TeV)	249.7	${}_{-4.4}^{+4.4}$	${}_{-6.3}^{+3.9}$	${}^{-2.5}_{+2.6}$	2.099
LHC (13 TeV)	816.2	${}_{-2.9}^{+2.9}$	${}_{-5.6}^{+3.5}$	${}^{-2.3}_{+2.4}$	1.681
Tevatron (1.96 TeV)	7.2	${}_{-3.1}^{+3.5}$	${}_{-5.5}^{+3.8}$	${}^{-2.7}_{+2.8}$	2.396
**CT14 (NNLO + NNLL)**					
LHC (7 TeV)	177.9	${}_{-3.7}^{+4.4}$	${}_{-3.5}^{+2.6}$	${}^{-2.6}_{+2.7}$	2.545
LHC (8 TeV)	253.6	${}_{-3.4}^{+3.9}$	${}_{-3.5}^{+2.6}$	${}^{-2.5}_{+2.6}$	2.459
LHC (13 TeV)	825.9	${}_{-2.7}^{+2.6}$	${}_{-3.6}^{+2.4}$	${}^{-2.3}_{+2.4}$	2.178
Tevatron (1.96 TeV)	7.4	${}_{-2.2}^{+3.5}$	${}_{-2.9}^{+1.6}$	${}^{-2.7}_{+2.8}$	1.842
**NNPDF30_nolhc (NNLO + NNLL)**					
LHC (7 TeV)	180.1	${}_{-5.0}^{+4.9}$	${}_{-3.5}^{+2.6}$	${}^{-2.6}_{+2.7}$	2.296
LHC (8 TeV)	256.7	${}_{-4.4}^{+4.3}$	${}_{-3.5}^{+2.6}$	${}^{-2.5}_{+2.6}$	2.147
LHC (13 TeV)	835.0	${}_{-2.8}^{+2.8}$	${}_{-3.6}^{+2.4}$	${}^{-2.3}_{+2.4}$	1.722
Tevatron (1.96 TeV)	7.3	${}_{-3.2}^{+3.6}$	${}_{-2.9}^{+1.5}$	${}^{-2.7}_{+2.8}$	2.476

**Fig. 1:**
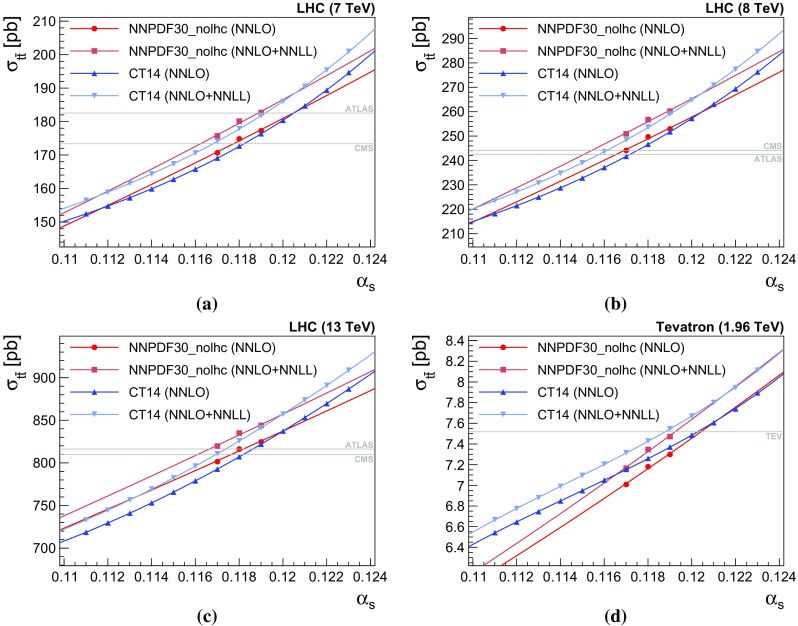
Predicted cross section as a function of $\alpha _s$. The points are the cross sections calculated using the Top++ program [23], and the line is our polynomial fit. The plot also includes horizontal lines corresponding to the central values of the measured cross sections, adjusted to correspond to the same top mass as the theory cross sections ($m_t = m_t^\text {ref} = 173.2\,\mathrm {GeV}$), cf. Sect. 2.4

We use PDF uncertainties calculated at the 68% confidence level, following the error propagation prescription from the individual PDF groups. The uncertainties from the CT14 PDF set, which are provided at a 90% confidence level by default, are scaled by a factor of $1/(\sqrt{2} \, \text {erf}^{-1}( 0.90) )\simeq 0.608$.

The predicted cross sections for both PDF sets, with NNLO and NNLO + NNLL calculations, are listed in Table 3. The cross sections are $1{-}3\%$ higher when including NNLL contributions. The scale uncertainties are in the $4{-}6\%$ range for the NNLO results and get reduced by between one third and one half when including NNLL terms. At LHC energies, the cross sections with NNPDF30_nolhc are about $1\%$ larger than those with CT14, however, the opposite pattern is seen at Tevatron. Finally, the PDF uncertainties are somewhat larger with NNPDF_nolhc than with CT14.

To understand the final errors on the $\alpha _s$ determination it is important also to examine how the predicted cross sections depend on $\alpha _s$, a result of the $\alpha _s$ dependence both of the hard cross section and of the PDFs. This is shown in Fig. 1: points correspond to the values of $\alpha _s$ for which the given PDF is available, and lines correspond to a fit for $\ln \sigma _{t\bar{t}}$ using a polynomial of $\ln \alpha _s$. We use polynomials of degree 3 and 1, respectively, for the CT14 and NNPDF30_nolhc PDFs, chosen based on the available number of $\alpha _s$ points and requirements of stability of the extrapolation beyond the available $\alpha _s$ points. A steeper slope of the $\alpha _s$ dependence (also quoted at $\alpha _s=0.118$ in the last column of Table 3) leads to a smaller final error on $\alpha _s$ for any given source of uncertainty on $\sigma _{t\bar{t}}$. For LHC energies, CT14 is generally steeper, while at the Tevatron it is NNPDF_nolhc that is steeper. Note also that CT14 curves have substantial curvature, and this will induce asymmetric uncertainties for $\alpha _s$, even in the case of uncertainties on the cross section that are symmetric.

### Top-mass dependence

The top-quark pole mass is taken to be $173.2 \pm 0.87\,$GeV, which is consistent with the world average value computed by the Particle Data Group [3]. The experimentally measured cross section, $\sigma _{t\bar{t}}^{\text {exp}} (m_t)$, depends on $m_t$ through the acceptance corrections, whose parametrisation is given together with the individual measurements. The uncertainty on the experimentally measured cross section due to the top-quark pole mass is given in Table 1, where the uncertainty was calculated by shifting the top mass up and down by its uncertainty. An increase in the top mass leads to a decrease in the measured total cross section. This is because the experiments effectively measure a fiducial cross section (which is independent of $m_t$) and then extrapolate it to a total cross section by dividing by the acceptance for the fiducial cross section. For larger values of $m_t$ the acceptance is larger, since decay products are more likely to pass transverse momentum cuts, and so the resulting total cross section is lower. The theoretically predicted cross section, $\sigma _{t\bar{t}}^{\text {pred}} ( m_t)$, also depends on $m_t$, because of the structure of the underlying hard cross section and the *x*-dependence of the PDFs, cf. Table 3. It too decreases for an increase in the cross section, and this effect is larger than for the measured cross section.

To define a single error contribution associated with the top-mass uncertainty, it is convenient to absorb these different sources of $m_t$ dependence into an effective predicted cross section,

2.1 $$\begin{aligned} \sigma _{t\bar{t}}^{\text {eff}}(m_t) = \sigma _{t\bar{t}}^{\text {pred}} ( m_t) \cdot \frac{ \sigma _{t\bar{t}}^{\text {exp}} \left( m_t^\text {ref}\right) }{ \sigma _{t\bar{t}}^{\text {exp}} ( m_t) }, \end{aligned}$$

where $m_t^\text {ref}= 173.2$ is the central value of the world average top mass. For $m_t = m_t^\text {ref}$, this effective predicted cross section coincides with the actual predicted one.

The final uncertainty on the effective predicted cross section associated with the error of $\Delta m_t = 0.87\,\mathrm {GeV}$ on the world average top mass is then given by

2.2 $$\begin{aligned} \sigma _{t\bar{t}}^{\text {eff}}\left( m_t^\text {ref}\pm \Delta m_t\right) - \sigma _{t\bar{t}}^{\text {eff}}\left( m_t^\text {ref}\right) . \end{aligned}$$

This can be used in our $\alpha _s$ determination in a manner similar to any of the theoretical and PDF uncertainties on the predicted cross section. To a good approximation, the final top-mass uncertainty on the effective cross section is equal to the difference between the percentage uncertainties in Tables 1 and 3.

### Strong coupling determination procedure

In the determination of $\alpha _s$ from $\sigma _{t\bar{t}}$, the theory prediction is treated as a Bayesian prior (one prior for any given value of $\alpha _s$) and the experimental result as a likelihood function. The multiplication of these is the joint posterior probability function from which $\alpha _s$ and its uncertainties are determined after marginalisation of $\sigma _{t\bar{t}}$. The procedure is mostly analogous to that used by the CMS Collaboration in Ref. [12].

The construction of the Bayesian prior from the theory dependence necessitates a single probability distribution function given all individual theory uncertainties. The three theory uncertainties are each interpreted as corresponding to an asymmetric Gaussian function:

2.3 $$\begin{aligned}&f^{\text {Unc. source}} \; (\sigma _{t\bar{t}}\,|\, \alpha _s)\nonumber \\&\quad = \left\{ \begin{array}{ll} \frac{1}{\sqrt{2\pi } \Delta _-} \, \text {e} \,^{-\frac{1}{2} \left( \frac{ \sigma _{t\bar{t}}- \sigma _{t\bar{t}}^{\text {pred}}(\alpha _s) }{ \Delta _- } \right) ^2 } &{} \quad \text {if} \; \sigma _{t\bar{t}}\le \sigma _{t\bar{t}}^{\text {pred}},\\ \frac{1}{\sqrt{2\pi } \Delta _+} \, \text {e} \,^{ -\frac{1}{2}\left( \frac{ \sigma _{t\bar{t}}- \sigma _{t\bar{t}}^{\text {pred}}(\alpha _s) }{ \Delta _+ } \right) ^2 } &{} \quad \text {if} \; \sigma _{t\bar{t}}> \sigma _{t\bar{t}}^{\text {pred}}, \end{array}\right. \end{aligned}$$

where $\sigma _{t\bar{t}}^{\text {pred}}(\alpha _s)$ is the predicted central value at a given value of $\alpha _s$, and $\Delta _{+(-)}$ is the positive (negative) uncertainty from a given theory uncertainty source. This function has the advantage that the integral normalises naturally to one, and that the integral from $(\sigma _{t\bar{t}}^{\text {pred}}-\Delta _-)$ to $(\sigma _{t\bar{t}}^{\text {pred}}+\Delta _+)$ corresponds to a 68% confidence interval. On average there is a 20% difference between $\Delta _+$ and $\Delta _-$, up to a difference of about 85% for the most asymmetric uncertainty. The central value for $\sigma _{t\bar{t}}$ corresponds to the median of the distribution.

The combined probability distribution function of the predicted cross section, $f^{\text {pred}}(\sigma _{t\bar{t}}\,|\,\alpha _s)$, is computed by taking the numerical convolution of the individual asymmetric Gaussian functions:

2.4 $$\begin{aligned} f^{\text {pred}}(\sigma _{t\bar{t}}\,|\,\alpha _s) = f^{\text {PDF}}(\sigma _{t\bar{t}}\,|\,\alpha _s) \otimes f^{m_t}(\sigma _{t\bar{t}}\,|\,\alpha _s) \otimes f^{\text {Scale}}(\sigma _{t\bar{t}}\,|\,\alpha _s),\nonumber \\ \end{aligned}$$

where the convolution is performed such that the probability distribution functions are centred around $\sigma _{t\bar{t}}^\text {pred}$. While the individual uncertainty distributions contain a discontinuity at $\sigma _{t\bar{t}}= \sigma _{t\bar{t}}^{\text {pred}}(\alpha _s)$, the convolution is a smooth function. The dependence on $\alpha _s$ of the width of the uncertainty band is neglected.^5^ The probability distribution function of the predicted cross section is multiplied by the probability distribution function of the measured cross section $f^{\text {exp}}(\sigma _{t\bar{t}}\,|\,\alpha _s)$, yielding the joint Bayesian posterior in terms of $\alpha _s$ and $\sigma _{t\bar{t}}$. The Bayesian confidence interval of $\alpha _s$ can be computed through marginalisation of the posterior by integrating over $\sigma _{t\bar{t}}$:

2.5 $$\begin{aligned} L(\alpha _s) = \int f^{\text {pred}}(\sigma _{t\bar{t}}\,|\,\alpha _s) \cdot f^{\text {exp}}(\sigma _{t\bar{t}}\,|\,\alpha _s) \; \text {d}\sigma _{t\bar{t}}. \end{aligned}$$

**Fig. 2:**
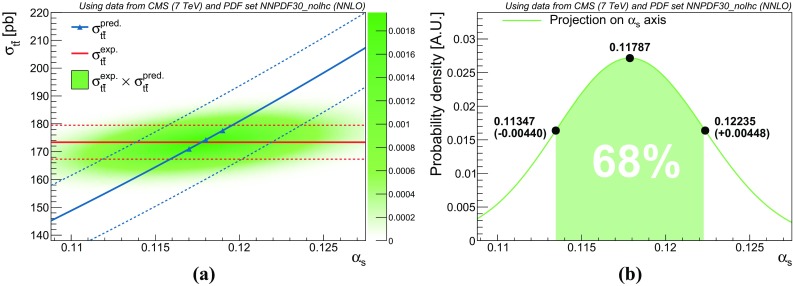
**a** The central values and 1$\sigma$ deviations for the predicted cross section ($f^{\text {pred}}(\sigma _{t\bar{t}}\,|\,\alpha _s)$, blue oblique lines) and the experimental cross section ($f^{\text {exp}}(\sigma _{t\bar{t}}\,|\,\alpha _s)$, red horizontal lines) and the product of the probability distribution functions (green shading). The markers on the predicted cross section indicate the fit points from top++2.0. **b** Marginalisation of the joint posterior with Bayesian confidence interval

Here, $f^{\text {exp}}(\sigma _{t\bar{t}}\,|\,\alpha _s)$ is taken to be independent of $\alpha _s$. Technically a small dependence on $\alpha _s$ is introduced in $f^{\text {exp}}(\sigma _{t\bar{t}}\,|\,\alpha _s)$ through the acceptance corrections; however, in the region of relevance around $\alpha _s^{\text {ref}} = 0.118$, the effect of this on the uncertainty of the cross section is below the percent level [12], and can thus be safely neglected. The marginalised joint posterior $L(\alpha _s)$ can be treated as a probability distribution function. The central value for the $\alpha _s$ determination is taken to be the location of the peak of $L(\alpha _s)$, and the uncertainty is extracted by computing the 68% confidence interval whose left and right bounds are at equal height.^6^ The procedure is illustrated in Fig. 2, showing the experimental and theory probability distribution functions and the unmarginalised posterior (Fig. 2a) as well as the marginalised posterior with extracted central value and uncertainties (Fig. 2b).

The combination of determinations from different experiments necessitates a breakdown of the total uncertainty into components that can be assigned to the individual uncertainty sources. To this end, the determination is repeated each time omitting a different uncertainty source, and the squared difference of the resulting uncertainty with respect to the total uncertainty is computed. A relative contribution to the total uncertainty is then computed per uncertainty source.

### Individual results for $\alpha _s$ per $\sigma _{t\bar{t}}$ measurement

The results of our $\alpha _s$ determination are listed for the CT14nnlo PDF set in Tables 4 and 6 and for the NNPDF30_nolhc PDF set in Tables 5 and 7.

The individual $\alpha _s$ determinations are all compatible with the world average to within uncertainties. The central values are rather similar to the CT14 and NNPDF sets. The largest individual sources of uncertainty on $\alpha _s$ are the PDF uncertainties and the scale uncertainties. For the LHC determinations, the PDF uncertainties tend to be larger with NNPDF, in part a consequence of the larger uncertainties in the cross section in Table 3. However, the other uncertainties are also larger with NNPDF, because of its weaker dependence on $\alpha _s$.

The NNLO + NNLL determinations all have smaller $\alpha _s$ results, consistent with the larger cross sections in Table 3. The scale uncertainties are also noticeably smaller. Other uncertainties are largely unchanged.

A final comment concerns the somewhat larger scale, $m_t$ and PDF uncertainties with the CT14 PDF for the CMS 7 TeV case as compared to the ATLAS 7 TeV case, or also ATLAS 8 TeV as compared to ATLAS 7 TeV. In general with the CT14 PDF, a smaller value of $\alpha _s$ corresponds to larger uncertainties, because the $\alpha _s$ dependence of the cross section is weaker for small $\alpha _s$ values; cf. Fig. 1. Note, however, that the scale and other uncertainties on the cross section predictions have been evaluated only for the reference value of $\alpha _s=0.118$, and in general the question of how one should correlate uncertainties with the central value is a delicate one.^7^ Accordingly one should be wary of reading too much into the variation of uncertainties with the central $\alpha _s$ value.

**Table 4:** Results for the strong coupling evaluated at the Z-boson mass scale and individual uncertainty contributions. These are based on cross sections calculated at NNLO using the CT14nnlo series of PDFs

	Center	Stat.	Syst.	Lumi.	$E_{\text {beam}}$	PDF	Scale	$m_t$	Total
ATLAS (7 TeV)	0.1205	${}_{-0.0009}^{+0.0007}$	${}_{-0.0012}^{+0.0009}$	${}_{-0.0010}^{+0.0008}$	${}_{-0.0001}^{+0.0001}$	${}_{-0.0021}^{+0.0015}$	${}_{-0.0021}^{+0.0021}$	${}_{-0.0012}^{+0.0009}$	${}_{-0.0036}^{+0.0030}$
ATLAS (8 TeV)	0.1171	${}_{-0.0004}^{+0.0003}$	${}_{-0.0014}^{+0.0011}$	${}_{-0.0013}^{+0.0010}$	${}_{-0.0002}^{+0.0001}$	${}_{-0.0025}^{+0.0017}$	${}_{-0.0026}^{+0.0027}$	${}_{-0.0015}^{+0.0011}$	${}_{-0.0044}^{+0.0037}$
ATLAS (13 TeV)	0.1187	${}_{-0.0006}^{+0.0006}$	${}_{-0.0021}^{+0.0017}$	${}_{-0.0014}^{+0.0012}$	${}_{-0.0001}^{+0.0001}$	${}_{-0.0016}^{+0.0014}$	${}_{-0.0024}^{+0.0026}$	${}_{-0.0013}^{+0.0011}$	${}_{-0.0041}^{+0.0038}$
CMS (7 TeV)	0.1182	${}_{-0.0007}^{+0.0005}$	${}_{-0.0014}^{+0.0010}$	${}_{-0.0013}^{+0.0009}$	${}_{-0.0002}^{+0.0001}$	${}_{-0.0025}^{+0.0017}$	${}_{-0.0025}^{+0.0025}$	${}_{-0.0014}^{+0.0010}$	${}_{-0.0043}^{+0.0035}$
CMS (8 TeV)	0.1175	${}_{-0.0004}^{+0.0003}$	${}_{-0.0015}^{+0.0011}$	${}_{-0.0016}^{+0.0012}$	${}_{-0.0001}^{+0.0001}$	${}_{-0.0024}^{+0.0017}$	${}_{-0.0026}^{+0.0026}$	${}_{-0.0014}^{+0.0010}$	${}_{-0.0044}^{+0.0037}$
CMS (13 TeV)	0.1183	${}_{-0.0007}^{+0.0006}$	${}_{-0.0030}^{+0.0025}$	${}_{-0.0015}^{+0.0013}$	${}_{-0.0001}^{+0.0002}$	${}_{-0.0017}^{+0.0014}$	${}_{-0.0025}^{+0.0026}$	${}_{-0.0010}^{+0.0009}$	${}_{-0.0047}^{+0.0042}$
Tevatron (1.96 TeV)	0.1202	${}_{-0.0018}^{+0.0013}$	${}_{-0.0026}^{+0.0019}$	${}_{-0.0019}^{+0.0014}$	${}_{-0.0000}^{+0.0000}$	${}_{-0.0020}^{+0.0014}$	${}_{-0.0027}^{+0.0024}$	${}_{-0.0009}^{+0.0006}$	${}_{-0.0050}^{+0.0039}$

**Table 5:** As in Table 4, but now using NNLO cross sections with the NNPDF30_nolhc series of PDFs

	Center	Stat.	Syst.	Lumi.	$E_{\text {beam}}$	PDF	Scale	$m_t$	Total
ATLAS (7 TeV)	0.1206	${}_{-0.0009}^{+0.0009}$	${}_{-0.0013}^{+0.0012}$	${}_{-0.0011}^{+0.0010}$	${}_{-0.0001}^{+0.0002}$	${}_{-0.0027}^{+0.0025}$	${}_{-0.0025}^{+0.0029}$	${}_{-0.0013}^{+0.0012}$	${}_{-0.0043}^{+0.0044}$
ATLAS (8 TeV)	0.1166	${}_{-0.0004}^{+0.0004}$	${}_{-0.0013}^{+0.0012}$	${}_{-0.0012}^{+0.0011}$	${}_{-0.0002}^{+0.0001}$	${}_{-0.0026}^{+0.0024}$	${}_{-0.0026}^{+0.0032}$	${}_{-0.0014}^{+0.0013}$	${}_{-0.0043}^{+0.0045}$
ATLAS (13 TeV)	0.1183	${}_{-0.0007}^{+0.0007}$	${}_{-0.0024}^{+0.0022}$	${}_{-0.0017}^{+0.0016}$	${}_{-0.0001}^{+0.0002}$	${}_{-0.0021}^{+0.0020}$	${}_{-0.0029}^{+0.0035}$	${}_{-0.0015}^{+0.0015}$	${}_{-0.0049}^{+0.0051}$
CMS (7 TeV)	0.1179	${}_{-0.0007}^{+0.0006}$	${}_{-0.0013}^{+0.0013}$	${}_{-0.0012}^{+0.0011}$	${}_{-0.0001}^{+0.0001}$	${}_{-0.0028}^{+0.0025}$	${}_{-0.0025}^{+0.0030}$	${}_{-0.0013}^{+0.0012}$	${}_{-0.0044}^{+0.0045}$
CMS (8 TeV)	0.1170	${}_{-0.0003}^{+0.0003}$	${}_{-0.0014}^{+0.0013}$	${}_{-0.0015}^{+0.0014}$	${}_{-0.0002}^{+0.0001}$	${}_{-0.0026}^{+0.0024}$	${}_{-0.0026}^{+0.0032}$	${}_{-0.0013}^{+0.0012}$	${}_{-0.0044}^{+0.0046}$
CMS (13 TeV)	0.1178	${}_{-0.0008}^{+0.0008}$	${}_{-0.0034}^{+0.0032}$	${}_{-0.0017}^{+0.0016}$	${}_{-0.0002}^{+0.0003}$	${}_{-0.0021}^{+0.0020}$	${}_{-0.0029}^{+0.0034}$	${}_{-0.0011}^{+0.0011}$	${}_{-0.0054}^{+0.0055}$
Tevatron (1.96 TeV)	0.1205	${}_{-0.0014}^{+0.0013}$	${}_{-0.0020}^{+0.0019}$	${}_{-0.0014}^{+0.0014}$	${}_{-0.0000}^{+0.0000}$	${}_{-0.0017}^{+0.0015}$	${}_{-0.0021}^{+0.0023}$	${}_{-0.0007}^{+0.0007}$	${}_{-0.0040}^{+0.0039}$

**Table 6:** As in Table. 4, but now using NNLO + NNLL cross sections with the CT14nnlo series of PDFs

	Center	Stat.	Syst.	Lumi.	$E_{\text {beam}}$	PDF	Scale	$m_t$	Total
ATLAS (7 TeV)	0.1192	${}_{-0.0009}^{+0.0007}$	${}_{-0.0012}^{+0.0010}$	${}_{-0.0010}^{+0.0008}$	${}_{-0.0001}^{+0.0001}$	${}_{-0.0021}^{+0.0016}$	${}_{-0.0014}^{+0.0012}$	${}_{-0.0012}^{+0.0010}$	${}_{-0.0033}^{+0.0027}$
ATLAS (8 TeV)	0.1158	${}_{-0.0004}^{+0.0004}$	${}_{-0.0014}^{+0.0011}$	${}_{-0.0013}^{+0.0011}$	${}_{-0.0002}^{+0.0001}$	${}_{-0.0025}^{+0.0019}$	${}_{-0.0018}^{+0.0016}$	${}_{-0.0015}^{+0.0012}$	${}_{-0.0040}^{+0.0032}$
ATLAS (13 TeV)	0.1175	${}_{-0.0006}^{+0.0005}$	${}_{-0.0020}^{+0.0018}$	${}_{-0.0014}^{+0.0012}$	${}_{-0.0001}^{+0.0001}$	${}_{-0.0016}^{+0.0014}$	${}_{-0.0017}^{+0.0017}$	${}_{-0.0013}^{+0.0012}$	${}_{-0.0037}^{+0.0033}$
CMS (7 TeV)	0.1168	${}_{-0.0007}^{+0.0006}$	${}_{-0.0015}^{+0.0011}$	${}_{-0.0013}^{+0.0010}$	${}_{-0.0002}^{+0.0001}$	${}_{-0.0026}^{+0.0019}$	${}_{-0.0017}^{+0.0014}$	${}_{-0.0015}^{+0.0012}$	${}_{-0.0040}^{+0.0031}$
CMS (8 TeV)	0.1162	${}_{-0.0004}^{+0.0003}$	${}_{-0.0015}^{+0.0012}$	${}_{-0.0016}^{+0.0013}$	${}_{-0.0002}^{+0.0001}$	${}_{-0.0024}^{+0.0018}$	${}_{-0.0018}^{+0.0016}$	${}_{-0.0014}^{+0.0011}$	${}_{-0.0040}^{+0.0032}$
CMS (13 TeV)	0.1171	${}_{-0.0007}^{+0.0006}$	${}_{-0.0029}^{+0.0025}$	${}_{-0.0015}^{+0.0013}$	${}_{-0.0002}^{+0.0001}$	${}_{-0.0017}^{+0.0015}$	${}_{-0.0018}^{+0.0017}$	${}_{-0.0011}^{+0.0009}$	${}_{-0.0043}^{+0.0038}$
Tevatron (1.96 TeV)	0.1188	${}_{-0.0017}^{+0.0014}$	${}_{-0.0025}^{+0.0021}$	${}_{-0.0018}^{+0.0015}$	${}_{-0.0000}^{+0.0000}$	${}_{-0.0020}^{+0.0015}$	${}_{-0.0013}^{+0.0011}$	${}_{-0.0009}^{+0.0007}$	${}_{-0.0043}^{+0.0035}$

**Table 7:** As in Table 4, but now using NNLO + NNLL cross sections with the NNPDF30_nolhc series of PDFs

	Center	Stat.	Syst.	Lumi.	$E_{\text {beam}}$	PDF	Scale	$m_t$	Total
ATLAS (7 TeV)	0.1190	${}_{-0.0009}^{+0.0009}$	${}_{-0.0012}^{+0.0012}$	${}_{-0.0011}^{+0.0010}$	${}_{-0.0001}^{+0.0001}$	${}_{-0.0026}^{+0.0025}$	${}_{-0.0015}^{+0.0016}$	${}_{-0.0013}^{+0.0012}$	${}_{-0.0037}^{+0.0036}$
ATLAS (8 TeV)	0.1152	${}_{-0.0004}^{+0.0004}$	${}_{-0.0013}^{+0.0012}$	${}_{-0.0012}^{+0.0011}$	${}_{-0.0001}^{+0.0001}$	${}_{-0.0025}^{+0.0024}$	${}_{-0.0017}^{+0.0018}$	${}_{-0.0014}^{+0.0013}$	${}_{-0.0037}^{+0.0037}$
ATLAS (13 TeV)	0.1168	${}_{-0.0007}^{+0.0007}$	${}_{-0.0023}^{+0.0022}$	${}_{-0.0016}^{+0.0015}$	${}_{-0.0002}^{+0.0002}$	${}_{-0.0020}^{+0.0019}$	${}_{-0.0020}^{+0.0022}$	${}_{-0.0015}^{+0.0015}$	${}_{-0.0043}^{+0.0043}$
CMS (7 TeV)	0.1163	${}_{-0.0006}^{+0.0006}$	${}_{-0.0013}^{+0.0012}$	${}_{-0.0011}^{+0.0011}$	${}_{-0.0001}^{+0.0001}$	${}_{-0.0027}^{+0.0026}$	${}_{-0.0016}^{+0.0016}$	${}_{-0.0013}^{+0.0012}$	${}_{-0.0038}^{+0.0037}$
CMS (8 TeV)	0.1155	${}_{-0.0003}^{+0.0003}$	${}_{-0.0013}^{+0.0013}$	${}_{-0.0014}^{+0.0014}$	${}_{-0.0001}^{+0.0001}$	${}_{-0.0025}^{+0.0024}$	${}_{-0.0017}^{+0.0017}$	${}_{-0.0013}^{+0.0012}$	${}_{-0.0038}^{+0.0037}$
CMS (13 TeV)	0.1163	${}_{-0.0007}^{+0.0008}$	${}_{-0.0032}^{+0.0031}$	${}_{-0.0016}^{+0.0015}$	${}_{-0.0002}^{+0.0002}$	${}_{-0.0020}^{+0.0019}$	${}_{-0.0020}^{+0.0022}$	${}_{-0.0011}^{+0.0011}$	${}_{-0.0048}^{+0.0047}$
Tevatron (1.96 TeV)	0.1194	${}_{-0.0013}^{+0.0013}$	${}_{-0.0019}^{+0.0018}$	${}_{-0.0014}^{+0.0013}$	${}_{-0.0000}^{+0.0000}$	${}_{-0.0017}^{+0.0016}$	${}_{-0.0010}^{+0.0010}$	${}_{-0.0007}^{+0.0007}$	${}_{-0.0034}^{+0.0033}$

**Table 8:** Correlated, uncorrelated and total luminosity uncertainties with respect to the top-quark-pair production cross section (in percentages) [40–46]

$\sqrt{s}$	Experiment	Corr.	Uncorr.	Total
7 TeV	ATLAS	0.46	1.72	1.78
	CMS	0.46	2.13	2.17
8 TeV	ATLAS	0.60	1.84	1.94
	CMS	0.68	2.50	2.59
13 TeV	ATLAS	0.36	2.29	2.32
	CMS	0.36	2.31	2.34

## Combination of $\alpha _s$ determinations

### Correlation coefficients

A combination of measurements can strongly depend on the assumed or calculated correlations [39]. It is therefore necessary to carefully evaluate the correlation coefficients used for the combination. In the case of $\alpha _s$ determinations many correlations can be reasonably motivated or computed. The correlation coefficients between individual measurements are motivated per uncertainty source.

1. *Statistical uncertainties* are considered uncorrelated for all experimental inputs.
2. *Systematic uncertainties* are considered fully correlated only for measurements obtained with the same detector. This concerns the measurements performed by CMS and ATLAS at different centre-of-mass energies.
3. *Uncertainties due to beam energy* are fully correlated between ATLAS and CMS and are taken to be correlated across energies. The beam-energy uncertainty at the Tevatron was tiny and is neglected, as outlined in the caption of Table 1.
4. *Uncertainties due to luminosity* are partially correlated between ATLAS and CMS. The correlated component of the luminosity uncertainty stems from the uncertainty on the bunch current density and similarities in the Van der Meer scan fit model.
  The correlated and uncorrelated uncertainties are estimated using the same principles as used for the top-quark-pair production cross section combinations between ATLAS and CMS at 7 and 8 TeV [40, 41], updated with the latest luminosity determinations [42–46]. The luminosity uncertainty (as a percentage of the top-quark-pair production cross section) is displayed in Table 8. The luminosity uncertainties on $\alpha _s$ are taken to have the same correlation coefficient.

The uncertainties on the predicted cross sections (due to the PDF, the top-quark mass and the renormalisation and factorisation scale) are generally strongly correlated. The combination result strongly depends on the assumed correlation structure of these theoretical uncertainties if included in the combination, which is usually not known precisely in particular for the scale uncertainty. We therefore adopt a different procedure: The individual results are simultaneously shifted up and down by their respective total theory uncertainties, and the combination is re-evaluated. The difference between the upper and lower bounds and the original combination is taken to be the (asymmetric) theoretical uncertainty.

The impact of the alternative procedure of including also the theory uncertainties within a single combination is discussed in Appendix A.

### Combining correlated measurements: likelihood-based approach

In order to combine the individual results, we opted for a likelihood-based approach [47].^8^ In this approach a global likelihood function is constructed from the probability distribution functions of individual determinations. Let us suppose we have $n_m$ measurements of the top cross section and associated determinations of $\alpha _s$. For each determination *i*, $\alpha _s{}_{,i}$, we have $n_u$ uncorrelated error components, each specific to that determination. The magnitude of the *k*th uncorrelated error for determination *i* is labelled $\Delta ^k_i$. We additionally have $n_c$ error components that are correlated across all determinations. For each of the correlated components, *j*, we introduce a nuisance parameter $\theta _j$ that is common across all measurements. Its impact on measurement *i* is governed by a coefficient $\delta _i^j$. The full set of $\theta _j$ will be denoted $\varvec{\theta }$.

The likelihood will be composed of a product of probability distribution functions (pdfs).^9^ For each nuisance parameter we will have one pdf, a Gaussian distribution with a standard deviation of one:

3.1 $$\begin{aligned} \text {pdf}_{\theta _j} = \frac{1}{\sqrt{2\pi }} e^{-\theta _j^2/2} . \end{aligned}$$

**Fig. 3:**
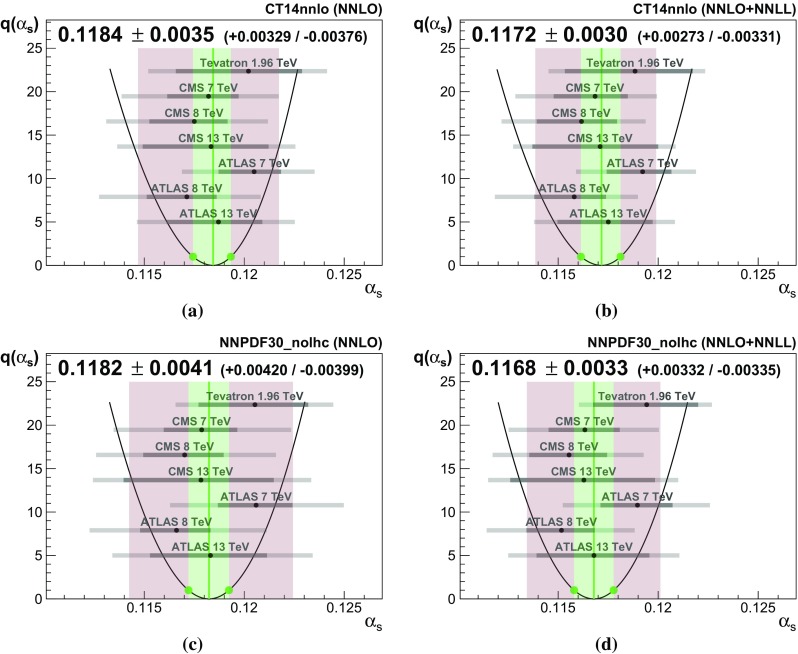
Combination results using the CT14 PDF set (NNLO in **a** and NNLO + NNLL in **b**) and the NNPDF3.0 noLHC PDF set (NNLO in **c** and NNLO + NNLL in **d**). The individual determinations and their uncertainties are shown in grey, where the darker shade represents the experimental uncertainties which enter into the combination. The test statistic *q* as a function of $\alpha _s$ is plotted as a black line. The green line and band represent the central value of the combination and the $1\sigma$ confidence interval, respectively. The red band depicts the total combination uncertainty with scale, PDF and top-mass uncertainties included

**Table 9:** Combination results for all PDF sets taken into consideration, at NNLO and NNLO + NNLL

	Center	Stat.	Syst.	$E_{\text {beam}}$	Lumi.	$m_t$	PDF	Scale	Total
CT14 (NNLO)	0.1184	${}_{-0.0003}^{+0.0003}$	${}_{-0.0007}^{+0.0006}$	${}_{-0.0001}^{+0.0001}$	${}_{-0.0006}^{+0.0006}$	${}_{-0.0014}^{+0.0010}$	${}_{-0.0023}^{+0.0016}$	${}_{-0.0025}^{+0.0025}$	${}_{-0.0038}^{+0.0033}$
NNPDF30_nolhc (NNLO)	0.1182	${}_{-0.0003}^{+0.0003}$	${}_{-0.0007}^{+0.0007}$	${}_{-0.0000}^{+0.0000}$	${}_{-0.0007}^{+0.0007}$	${}_{-0.0013}^{+0.0012}$	${}_{-0.0025}^{+0.0023}$	${}_{-0.0026}^{+0.0031}$	${}_{-0.0040}^{+0.0042}$
CT14 (NNLO + NNLL)	0.1172	${}_{-0.0003}^{+0.0003}$	${}_{-0.0007}^{+0.0007}$	${}_{-0.0001}^{+0.0001}$	${}_{-0.0007}^{+0.0006}$	${}_{-0.0014}^{+0.0011}$	${}_{-0.0023}^{+0.0017}$	${}_{-0.0017}^{+0.0015}$	${}_{-0.0033}^{+0.0027}$
NNPDF30_nolhc (NNLO + NNLL)	0.1168	${}_{-0.0003}^{+0.0003}$	${}_{-0.0007}^{+0.0006}$	${}_{-0.0001}^{+0.0001}$	${}_{-0.0007}^{+0.0007}$	${}_{-0.0013}^{+0.0012}$	${}_{-0.0024}^{+0.0023}$	${}_{-0.0017}^{+0.0018}$	${}_{-0.0034}^{+0.0033}$
Average	0.1177	${}_{+0.0003}^{+0.0003}$	${}_{+0.0007}^{+0.0007}$	${}_{+0.0001}^{+0.0001}$	${}_{+0.0007}^{+0.0006}$	${}_{+0.0013}^{+0.0012}$	${}_{+0.0024}^{+0.0020}$	${}_{+0.0021}^{+0.0022}$	${}_{-0.0036}^{+0.0034}$

There will also be a pdf for each combination of measurement *i* and associated uncorrelated error $\Delta ^k_i$. It is given by

3.2 $$\begin{aligned} \text {pdf}_{i,\,k}(\alpha _s, \varvec{\theta }) = \frac{1}{\sqrt{2\pi } \Delta _i^k} \, \exp { \displaystyle \left[ -\frac{ (\alpha _s{}_{,i} \, + \sum _j \theta _j \cdot \delta _i^j - \alpha _s)^2 }{ 2(\Delta _i^k)^2 } \right] } \;. \end{aligned}$$

To address the issue of errors that are not symmetric, we adopt the following prescription for the $\Delta _i^k$ and $\delta _i^j$:

3.3 $$\begin{aligned} \Delta _i^k= & {} \left\{ \begin{array}{ll} \displaystyle \Delta _i^{k,\,-} &{} \quad \text {if} \;\; \alpha _s\le \alpha _s{}_{,i},\\ \displaystyle \Delta _i^{k,\,+} &{} \quad \text {if} \;\; \alpha _s> \alpha _s{}_{,i},\end{array}\right. \;, \end{aligned}$$

3.4 $$\begin{aligned} \delta _i^j= & {} \left\{ \begin{array}{ll} \displaystyle \delta _i^{j,\,-}&{} \quad \text {if} \;\; \alpha _s\le \alpha _s{}_{,i}, \\ \displaystyle \delta _i^{j,\,+}&{} \quad \text {if} \;\; \alpha _s> \alpha _s{}_{,i} \end{array}\right. \;. \end{aligned}$$

An overview of the values used for $\delta _i^{j,\,\pm }$ and $\Delta _i^{k,\,\pm }$ is given in Appendix B. The probability distribution function of determination *i* including all uncorrelated uncertainties is then constructed by convolution:

3.5 $$\begin{aligned}&\text {pdf}_{ \alpha _s{}_{,i} }(\alpha _s, \varvec{\theta })\nonumber \\&\quad = \text {pdf}_{i,\,1}(\alpha _s, \varvec{\theta }) \otimes \text {pdf}_{i,\,2}(\alpha _s, \varvec{\theta }) \otimes \cdots \otimes \text {pdf}_{i,\,n_u}(\alpha _s, \varvec{\theta })\nonumber \\ \end{aligned}$$

where the convolution is performed such that the probability distribution functions are centred around $\alpha _s{}_{,i}$. The global likelihood function $L( \alpha _s, \varvec{\theta } )$ is constructed by multiplication of the probability distribution functions of the determinations and the nuisance parameters:

3.6 $$\begin{aligned} L( \alpha _s, \varvec{\theta } ) = \prod _{i=1}^{n_m} \text {pdf}_{ \alpha _s{}_{,i} }(\alpha _s, \varvec{\theta }) \, \times \, \prod _{j=1}^{n_c} \text {pdf}_{\theta _j} . \end{aligned}$$

In order to complete the formalism of a statistical test the test statistic *q* is introduced:

3.7 $$\begin{aligned} q( \alpha _s) = -2 \log \frac{ L( \alpha _s,\, \hat{\varvec{\theta }}' ) }{ L( \hat{\alpha }_s ,\, \hat{\varvec{\theta }} ) }. \end{aligned}$$

Here *L* is maximised for variables that carry a hat and in general $\hat{\varvec{\theta }}'$ will take on different values from $\hat{\varvec{\theta }}$. The quantity $L( \hat{\alpha }_s ,\, \hat{\varvec{\theta }} )$ is therefore the global maximum likelihood, and the ratio cannot be larger than one. The normalisation is such that *q* can be treated as $\chi ^2$-distributed with one degree of freedom.

**Fig. 4:**
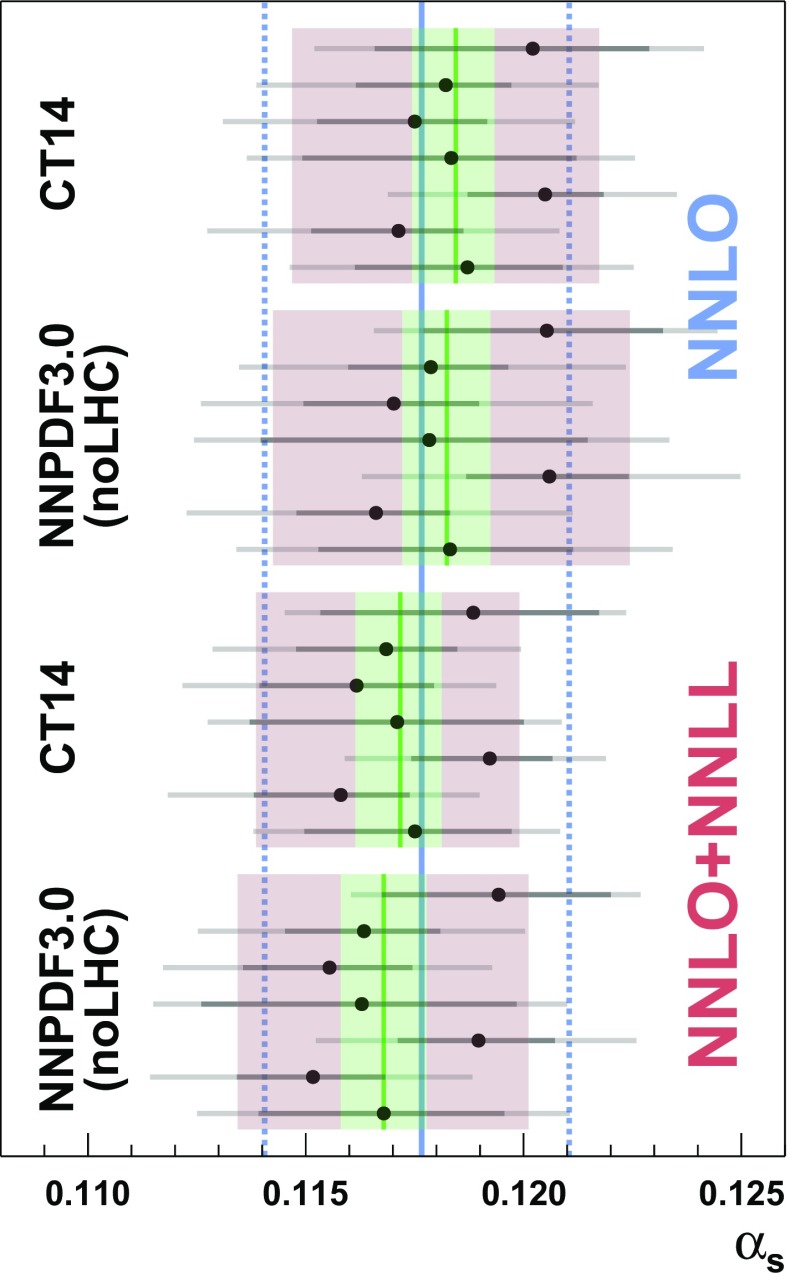
Combination results for all PDF sets taken into consideration, at NNLO and NNLO + NNLL. The solid blue line is the unweighted average of the individual combination results, and the dashed blue lines represent the 68% confidence interval. The red and green bands are as in Fig. 3

The test statistic *q* is scanned over a range of $\alpha _s$ values. The minimum of the scan, by construction at $q=0$, is the maximum likelihood value for $\alpha _s$, and the $1\sigma$ confidence interval is extracted from the interval between the intersection points of the scan with $q=1$. Any skewness of the parabola of the scan is due to the inclusion of asymmetric uncertainties. Figure 3 shows the scan and the corresponding combination results for each of the PDF sets.

## Results and discussion

The combination procedure is performed for each of the two PDF sets taken into consideration at NNLO and at NNLO + NNLL separately. The combination results and their unweighted average are displayed numerically in Table 9, and graphically in Fig. 4.

There is no unique way to quote a final best estimate of $\alpha _s$ based on the results obtained from the different PDF sets and QCD calculation choices (NNLO v. NNLO + NNLL). An unbiased approach for combining results from different PDFs, in line with the PDF4LHC recommendations [49], is to average without applying any further weighting. In accordance with that approach we take the straight average of the mean values and the uncertainties of the individual combinations. This coincides with the procedure for combining $\alpha _s$ results from a single class of observables in Ref. [3]. The final result is4.1which can be compared to the result of Ref. [12], $\alpha _s(m_Z) = 0.1151^{+0.0028}_{-0.0027}$. Our central value is larger mainly because recent measurements of the cross sections are higher than that used in Ref. [12], but also in part because of our choice to take the average of results from NNLO and NNLO + NNLL cross sections (a $0.6\%$ increase relative to just NNLO + NNLL). Our symmetrised uncertainty of $3.0\%$ is somewhat increased with respect to that of Ref. [12], $2.4\%$ (symmetrised). The difference in uncertainty is due to several choices. On one hand we have taken a smaller uncertainty on the top-quark mass, in line with the PDG determination. One the other hand, we have been somewhat more conservative in our treatment of theoretical and PDF uncertainties. Firstly, the choice of treating the scale uncertainties on $\sigma _{t\bar{t}}$ as a $68\%$ confidence interval instead of a (flat) $100\%$ confidence interval increases the scale uncertainty component by roughly a factor of $\sqrt{3}$. Secondly, we have used an average of the uncertainties from NNLO and NNLO + NNLL cross section determinations, which also yields a larger uncertainty than using NNLO + NNLL cross section determinations only. Finally, the PDF sets used for the determination were chosen with minimisation of potential biases in mind, rather than the ones with smallest uncertainty.

## Conclusions

We have used seven measurements of the top–antitop-quark production cross section at the LHC and the Tevatron in order to determine the strong coupling constant $\alpha _s \left( m_Z \right)$, using the CT14 PDF set and the NNPDF30_nolhc PDF set at NNLO and NNLO + N NLL. Overall, our determination of $\alpha _s$ yields a value that is compatible with the world average value and uncertainties that are somewhat larger than the best individual determinations, though comparable with that from the electroweak precision data [50]. The largest uncertainties are associated with unknown higher-order contributions and PDF uncertainties.

## A Including strongly correlated uncertainty sources in the combination

Our approach of excluding strongly correlated uncertainties from the combination is generally recommended when using the covariance matrix to fit strongly correlated data [51]. To illustrate the effect of strong correlations, the combination is here performed again with the PDF, scale and $m_t$ uncertainties included in the combination one by one. The PDF and scale uncertainties are considered fully correlated between measurements made with the LHC, and partially correlated between measurements made with the LHC and Tevatron. In the case of the PDF uncertainties the degree of correlation between LHC and Tevatron measurements was determined using the procedure described in Ref. [33]. The $m_t$ uncertainties are considered fully correlated for all measurements.

**Table 10:** Combination results including also uncertainties from the PDF, the scale and the top mass in the combination. The first row corresponds to our approach of excluding correlated uncertainties from the combination. The results listed here are obtained using NNLO cross sections with the CT14nnlo series of PDFs

Uncertainties included in combination	Center	Combination uncertainty	Total uncertainty
–	0.1184	${}_{-0.0010}^{+0.0009}$	${}_{-0.0038}^{+0.0033}$
PDF	0.1191	${}_{-0.0018}^{+0.0018}$	${}_{-0.0034}^{+0.0033}$
PDF and $m_t$	0.1194	${}_{-0.0020}^{+0.0020}$	${}_{-0.0032}^{+0.0032}$
PDF, $m_t$ and scale	0.1207	${}_{-0.0029}^{+0.0030}$	${}_{-0.0029}^{+0.0030}$

**Table 11:** As in Table 10, but now using NNLO + NNLL cross sections with the CT14nnlo series of PDFs

Uncertainties included in combination	Center	Combination uncertainty	Total uncertainty
–	0.1172	${}_{-0.0010}^{+0.0010}$	${}_{-0.0033}^{+0.0027}$
PDF	0.1180	${}_{-0.0020}^{+0.0019}$	${}_{-0.0029}^{+0.0027}$
PDF and $m_t$	0.1183	${}_{-0.0022}^{+0.0022}$	${}_{-0.0027}^{+0.0027}$
PDF, $m_t$ and scale	0.1188	${}_{-0.0025}^{+0.0025}$	${}_{-0.0025}^{+0.0025}$

**Table 12:** As in Table 10, but now using NNLO cross sections with the NNPDF30_nolhc series of PDFs

Uncertainties included in combination	Center	Combination uncertainty	Total uncertainty
–	0.1182	${}_{-0.0010}^{+0.0010}$	${}_{-0.0040}^{+0.0042}$
PDF	0.1188	${}_{-0.0022}^{+0.0023}$	${}_{-0.0037}^{+0.0040}$
PDF and $m_t$	0.1190	${}_{-0.0024}^{+0.0025}$	${}_{-0.0036}^{+0.0040}$
PDF, $m_t$ and scale	0.1200	${}_{-0.0036}^{+0.0035}$	${}_{-0.0036}^{+0.0035}$

**Table 13:** As in Table 10, but now using NNLO + NNLL cross sections with the NNPDF30_nolhc series of PDFs

Uncertainties included in combination	Center	Combination uncertainty	Total uncertainty
–	0.1168	${}_{-0.0010}^{+0.0010}$	${}_{-0.0034}^{+0.0033}$
PDF	0.1175	${}_{-0.0023}^{+0.0023}$	${}_{-0.0031}^{+0.0031}$
PDF and $m_t$	0.1178	${}_{-0.0025}^{+0.0025}$	${}_{-0.0030}^{+0.0030}$
PDF, $m_t$ and scale	0.1182	${}_{-0.0028}^{+0.0028}$	${}_{-0.0028}^{+0.0028}$

Tables 10 and 11 show the results for the CT14 PDF set, using NNLO and NNLO + NNLL, respectively, and Tables 12 and 13 for the NNPDF30_nolhc PDF set. As expected, the total uncertainty decreases as more sources are included in the combination. As the sensitivity to $\alpha _s$ is stronger for a larger cross section, determinations that deviate up can have a smaller uncertainty, and therefore obtain a larger weight in the combination. This is the case for the determination from the ATLAS measurement at 7 TeV when using the CT14 PDF set. A larger weight may also be obtained for determinations that are more independent with respect to the others. This is primarily the case for the Tevatron determination, for both PDF sets. These effects are enhanced if the overall correlation is increased by including strongly correlated uncertainty sources, which explains why the combination yields increasing values of $\alpha _s$ as more sources are included. The results found this way are larger than both the straight average and the median of the individual determinations, though the difference is well within one standard deviation. Taking them as our final results would imply a high degree of trust in the assumed correlations. Due to the inherent difficulty of determining correlations, notably as concerns the scale variations, and the importance of the subtle interplay between an individual determination’s $\alpha _s$ result and its error, the conservative approach is to exclude the strongly correlated sources from the combination.

## B Overview of asymmetric uncertainties used in the combination

Tables 14 and 15 show the numerical values for the uncertainty coefficients used in the combination procedure for the CT14 PDF set, using NNLO and NNLO + NNLL cross sections, respectively. Only experimental uncertainties are listed. Theoretical uncertainties, which are taken into account after the combination procedure, can be found in Tables 4, 5, 6 and 7. The correlations for the correlated uncertainties (with a $\delta$ symbol) are described in Sect. 3.1.

**Table 14:** Overview of the uncertainty coefficients for the CT14 (NNLO) PDF set. The coefficients with a $\delta$ correspond to the coefficients of the correlated uncertainty sources, and those with a $\Delta$ to the uncorrelated uncertainty sources

Exp.	$\delta ^{\text {Syst.}}$	$\delta ^{\text {Lumi.}}$	$\delta ^{\text {E}_{beam}}$	$\Delta ^{\text {Stat.}}$	$\Delta ^{\text {Lumi.}}$
ATLAS (13 TeV)	${}_{-0.0021}^{+0.0017}$	${}_{-0.0002}^{+0.0002}$	${}_{-0.0001}^{+0.0001}$	${}_{-0.0006}^{+0.0006}$	${}_{-0.0014}^{+0.0012}$
ATLAS (8 TeV)	${}_{-0.0014}^{+0.0011}$	${}_{-0.0004}^{+0.0003}$	${}_{-0.0002}^{+0.0001}$	${}_{-0.0004}^{+0.0003}$	${}_{-0.0013}^{+0.0009}$
ATLAS (7 TeV)	${}_{-0.0012}^{+0.0009}$	${}_{-0.0003}^{+0.0002}$	${}_{-0.0001}^{+0.0001}$	${}_{-0.0009}^{+0.0007}$	${}_{-0.0010}^{+0.0007}$
CMS (13 TeV)	${}_{-0.0030}^{+0.0025}$	${}_{-0.0002}^{+0.0002}$	${}_{-0.0001}^{+0.0002}$	${}_{-0.0007}^{+0.0006}$	${}_{-0.0015}^{+0.0013}$
CMS (8 TeV)	${}_{-0.0015}^{+0.0011}$	${}_{-0.0004}^{+0.0003}$	${}_{-0.0001}^{+0.0001}$	${}_{-0.0004}^{+0.0003}$	${}_{-0.0016}^{+0.0012}$
CMS (7 TeV)	${}_{-0.0014}^{+0.0010}$	${}_{-0.0003}^{+0.0002}$	${}_{-0.0002}^{+0.0001}$	${}_{-0.0007}^{+0.0005}$	${}_{-0.0013}^{+0.0009}$
Tevatron (1.96 TeV)	${}_{-0.0026}^{+0.0019}$	–	–	${}_{-0.0018}^{+0.0013}$	${}_{-0.0019}^{+0.0014}$

**Table 15:** As in Table 14, but now using NNLO + NNLL cross sections with the CT14 PDF set

Exp.	$\delta ^{\text {Syst.}}$	$\delta ^{\text {Lumi.}}$	$\delta ^{\text {E}_{beam}}$	$\Delta ^{\text {Stat.}}$	$\Delta ^{\text {Lumi.}}$
ATLAS (13 TeV)	${}_{-0.0020}^{+0.0018}$	${}_{-0.0002}^{+0.0002}$	${}_{-0.0001}^{+0.0001}$	${}_{-0.0006}^{+0.0005}$	${}_{-0.0014}^{+0.0012}$
ATLAS (8 TeV)	${}_{-0.0014}^{+0.0011}$	${}_{-0.0004}^{+0.0003}$	${}_{-0.0002}^{+0.0001}$	${}_{-0.0004}^{+0.0004}$	${}_{-0.0013}^{+0.0010}$
ATLAS (7 TeV)	${}_{-0.0012}^{+0.0010}$	${}_{-0.0003}^{+0.0002}$	${}_{-0.0001}^{+0.0001}$	${}_{-0.0009}^{+0.0007}$	${}_{-0.0010}^{+0.0008}$
CMS (13 TeV)	${}_{-0.0029}^{+0.0025}$	${}_{-0.0002}^{+0.0002}$	${}_{-0.0002}^{+0.0001}$	${}_{-0.0007}^{+0.0006}$	${}_{-0.0015}^{+0.0013}$
CMS (8 TeV)	${}_{-0.0015}^{+0.0012}$	${}_{-0.0004}^{+0.0003}$	${}_{-0.0002}^{+0.0001}$	${}_{-0.0004}^{+0.0003}$	${}_{-0.0016}^{+0.0012}$
CMS (7 TeV)	${}_{-0.0015}^{+0.0011}$	${}_{-0.0003}^{+0.0002}$	${}_{-0.0002}^{+0.0001}$	${}_{-0.0007}^{+0.0006}$	${}_{-0.0013}^{+0.0010}$
Tevatron (1.96 TeV)	${}_{-0.0025}^{+0.0021}$	–	–	${}_{-0.0017}^{+0.0014}$	${}_{-0.0018}^{+0.0015}$

## Footnotes

1 An alternative analysis of the thrust quotes a significantly larger uncertainty, $0.1137^{+0.0034}_{-0.0027}$ [10].

2 As discussed in [27] and also [28]. Note that the experience with NLO scale uncertainties may not apply to NNLO scale uncertainties. In particular, for the two cases of hadron-collider calculations available at N3LO accuracy, Higgs production in the gluon-fusion [4] and vector-boson-fusion [29] channels, while the central NNLO results are outside the NLO scale uncertainty bands, the N3LO results are well within the corresponding NNLO bands.

3 The $\sigma _{t\bar{t}}$ measurement by CMS at 13 TeV using events with one lepton and at least one jet in the final state [15] has a slightly better precision than the CMS result used in our analysis. However, the effect on the final result is marginal, and using measurements from the same decay channel yields a clearer correlation structure for the combination.

4 As this article was being completed the NNPDF31 series [38] of PDF sets became available. It includes a set fitted without top data, however, only for a single value of the strong coupling, and accordingly is not suitable for use in a strong coupling determination.

5 With this approach of fixed absolute uncertainties on $\sigma _{t\bar{t}}$, theory uncertainties on $\alpha _s$ will turn out relatively smaller for determinations with a higher central $\alpha _s$ value. One concern is that this might affect the relative weights of different determinations in the combination that is described later in Sect. 3. To address this concern, a cross-check was performed in which the individual theory errors from our procedure were scaled relative to the default approach by a factor $\frac{ \alpha _s^\text {determination} }{ \alpha _s^\text {ref} }$. That is equivalent to taking fixed relative (rather than fixed absolute) theory uncertainties on $\sigma _{t\bar{t}}$. With the combination procedure of Sect. 3, the difference induced by this change was below the per mille level. For the alternative combination procedure in Appendix A, the effect is less than half a percent on $\alpha _s$, which remains much smaller than the difference between the two combination procedures.

6 This is somewhat different from the prescription to define an asymmetric probability distribution in Eq. (2.3), but it coincides with widespread practice in ATLAS and CMS likelihood fits.

7 As an example, imagine that we had used scale uncertainties that depended on $\alpha _s$: then for an experimental measurement with cross section that fluctuates low, one would deduce a smaller scale uncertainty than for a cross section that fluctuates high; when combining them, depending on the procedure, this might then lead to a larger weight for the smaller value of $\alpha _s$.

8 As a cross-check, we also used the *Best Linear Unbiased Estimate* procedure (BLUE) [39, 48]. This is only suitable for symmetric errors and in that case we found essentially identical results.

9
*pdf*, for probability density function, is not to be confused with *PDF*, for parton distribution function.
